# Major Climate risks and Adaptation Strategies of Smallholder Farmers in Coastal Bangladesh

**DOI:** 10.1007/s00267-020-01291-8

**Published:** 2020-05-09

**Authors:** Jeetendra Prakash Aryal, Tek Bahadur Sapkota, Dil Bahadur Rahut, Timothy J. Krupnik, Sumona Shahrin, M. L. Jat, Clare M. Stirling

**Affiliations:** 1 International Maize and Wheat Improvement Center (CIMMYT), Carretera México-Veracruz Km. 45 El Batán, Texcoco, Mexico; 2 International Maize and Wheat Improvement Center (CIMMYT), New Delhi, India; 3 International Maize and Wheat Improvement Center (CIMMYT), Dhaka, Bangladesh; 4 Global R&D Technology Lead, Cocoa Life, Mondelez International, Birmingham, UK

**Keywords:** Gender, Climate change adaptation, Climate risks, Smallholder farmers, Multivariate probit model

## Abstract

Rural households in South Asia’s coastal deltas face numerous livelihood challenges, including risks posed by climatic variability and extreme weather events. This study examines major climate risks, farmers’ adaptation strategies, and the factors affecting the choice of those strategies using data collected from 630 households in southwestern coastal Bangladesh. Farmers identified cyclones, excessive rain and flooding, and salinity as direct climate risks. Increased crop diseases/pests and livestock diseases were perceived as indirect risks resulting from climatic variability. Farmers used multiple adaptation strategies against those risks such as modifications in farm management, use of savings and borrowing funds from family and neighbors, and periodically reducing household food consumption. Off-farm employment and seeking assistance from governmental as well as non-governmental organizations (NGOs) were also common adaptation strategies. The results show that male-headed households are more likely to change farming practices and reduce consumption compared with female-headed households that conversely tended to take assistance from NGOs as an adaptation strategy. Ownership of land and livestock, as well as farmers’ prior exposure to climate change and educational training, also had a significant effect on the choice of adaptation strategy. Therefore, development interventions and policies that aimed at improving resource endowment and training to farmers on climatic risks and their adaptation strategies can help minimize the impact of climatic risks.

## Introduction

Climate-related risks, including extreme events such as cyclones, excessive rainfall, and consequent flooding and waterlogging, soil salinity, and river bank erosion, have been widely acknowledged to negatively affect rural livelihoods in South Asia’s coastal regions (Dastagir 2015; Karim and Mimura 2008). The geographical location of Bangladesh with its relatively low-lying, flat topography, renders it one of the most vulnerable countries in the world to climate risks (IPCC 2007). Without adaptation and improvements in coastal embankment systems, a one-meter rise in sea level resulting from longer-term climate change could flood ~18% of the country’s land area (Khan et al. 2010). Riverine flooding and waterlogging resulting from high-intensity rainfall events also adversely affect the livelihood of rural communities (Ruane et al. 2013; Thomas et al. 2013). With a 32 cm rise in sea level, and consequent salinization processes, the area suitable for the cultivation of rainfed “aman” rice (i.e., the main season rice) that provides most of the calories consumed in Bangladesh could decline by up to 60% (Pender 2008). Almost six million people are already exposed to soil and water salinity in the coastal region, which is affected by upstream water diversions and can be accelerated with sea-level rise and climate change (Krupnik et al. 2017). By 2050 and 2080, unchecked progress in salinity could affect the life and livelihood of 13.6 and 14.8 million people, respectively (Khan et al. 2010). Extreme weather events are predicted to become more frequent and intense in the future, with potentially serious negative consequences on the livelihood of millions of farmers in Bangladesh (Dastagir 2015; Dewan 2015; IPCC 2007; Karim and Mimura 2008).

Impacts of these climatic risks are particularly severe for smallholder farmers that make up the bulk of the rural population in Bangladesh. As the agriculture sector contributes 13% of the country’s gross domestic product and employs 48% of the labor force (World Bank 2019), adaptation to climatic risks requires special attention. In addition to the immediate problems associated with developing more climate-resilient agricultural systems, institutional inefficiencies, poorly developed infrastructure, and the region’s generally high population pressure pose additional development challenges. The degree to which rural communities are vulnerable to these risks not only depends on their initial severity, but also on secondary effects including new pests and diseases that result from changes in the climate, in addition to the adaptive capacity of the farming community (Baker et al. 2012). Given that farmers can use several strategies to deal with climate risks, in this study, we examine the major climate risks faced by farmers in the southwestern coastal region of Bangladesh and discuss major adaptation strategies they adopt to minimize vulnerability.

Though climate change affects all farmers, it is expected to disproportionately affect poor and marginalized communities that depend entirely on agriculture for their livelihoods and who have a low level of resource endowment and capacity to adapt to such changes (FAO 2012; World Bank 2011). Female farmers in developing countries are most vulnerable to climate risk due to their low capacity to adapt arising from limited access to livelihood assets such as financial, physical, social, and human capital. These effects may also be important to understand the adaptation decisions made by farmers, and the ways in which farmers with different levels of assets, livelihood strategies, and how men and women differentially mitigate climate risks. Review of the impacts of climate change on major cereal crops in Bangladesh shows that it is generally negative, and thus, adaptation to climate change is crucial to reduce the vulnerability of the farming communities (Aryal et al. 2019). Trans-disciplinary studies on climate change adaptation are proposed, with emphasis on socially-relevant topics that can affect public policy because much of the available literature focuses on economic considerations, with the less comprehensive literature on the environmental and social consequences of climate change (Rahman et al. 2018).

In addressing these issues, an understanding of the ways in which male and female-headed households choose adaptation strategies is equally crucial as it has implications for development programs that improve access to resources and information with the goal of socially equitable development (Bryan et al. 2009; Deressa and Hassan 2009; Deressa et al. 2011, 2009; Partey et al. 2020; World Bank 2012). The socially constructed role of women as primary domestic providers in Bangladesh exerts a strong influence on these challenges, as women’s liberty to appear in public, migrate, own property, or make agricultural decisions can render them more vulnerable to climate shocks and disasters, and gendered experience of climate stress (Dilley et al. 2005; Jordan 2019; Reggers 2019). In Bangladesh, women are disproportionally affected by extreme climatic events, including cyclones (Kabir et al. 2016). Further, gender norms may also restrict women from adapting to climate risks. Owing to different experiences, perspectives, and social capital, men and women’s livelihoods and adaptation strategies are also likely to be different (Akter et al. 2016; Corcoran-Nantes and Roy 2018; Reggers 2019). Besides the differential access to resources, the ability to take hold of livelihood diversification opportunities influences the adaptive capacity of men and women (Deressa et al. 2009; Djoudi and Brockhaus 2011; Partey et al. 2020).

In response to these issues, we utilized data from 630 farm households across 12 villages in Bagerhat, Jhalokathi, and Satkhira districts in southwestern Bangladesh, to study perceptions of the major climate risks faced by farmers and adaptation strategies applied to minimize the vulnerability due to those risks, and factors explaining the choice of adaptation strategies by farmers. Such a study is important for designing development interventions in Bangladesh, where the majority of the farmers are smallholders and poor.

## Study Area and Data

### Study Area

**Fig. 1:**
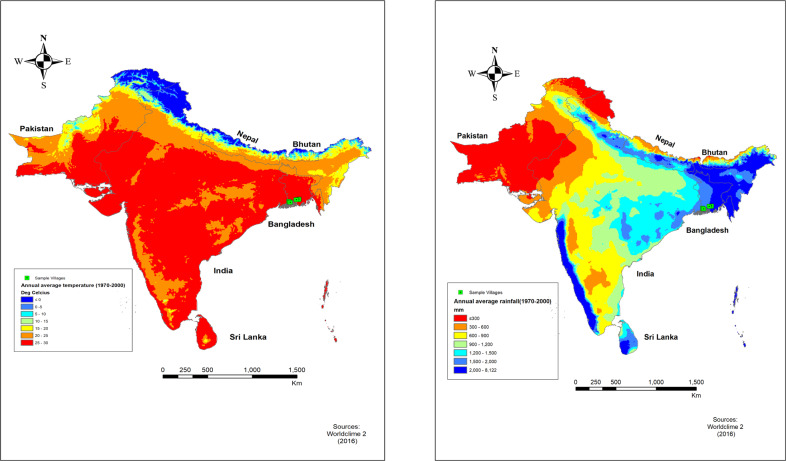
Regional climate map in relation to study sites

This study focused on Jhalokathi, Bagerhat, and Satkhira districts in Barisal and Khulna division in south western Bangladesh (see Fig. 1). Figure 1 shows the study sites in Bangladesh in the context of heat and rainfall at the regional level. From the map, it is clear that the study sites from heavy rainfall and high temperature, making it vulnerable to climate risk. The vulnerability of the population living in the study area to climate risk is exacerbated due to the fact that it is very close to the sea. In the light of the climate extreme facing the household in the coastal area of Bangladesh, it is of paramount importance to focus on understanding the climate risk and adaptation strategies available and in practice in the area.

A large proportion of the population in Bagerhat is involved in agriculture as a primary source of livelihoods (BCAS 2013). Rice is the major crop produced in this area (Bangladesh Bureau of Statistics 2012). Of the three districts under study, Satkhira district is exposed to relatively high levels of salinity in both soil and water during the dry season, as compared with Bagerhat and Jhalokati (Braun and Saroar 2012). In addition to climate change, upstream diversions are likely to exacerbate salinity problems in the future in Satkhira and Bagerhat districts (Mohal and Hossain 2007). Salinity intrusion already negatively affected rice production in Satkhira district (Rabbani et al. 2013). The study area is prone to extreme weather events, including heavy pre-monsoon season storms and cyclones, and consequential waterlogging (Ali 2007). Bagerhat and Satkhira districts are also relatively more vulnerable in terms of food insecurity and poverty (Dasgupta et al. 2014).

### Data Collection and Survey Methods

Data were obtained from a household survey conducted by the International Maize and Wheat Improvement Centre (CIMMYT) in 2013 as part of the CGIAR Research Program on Climate Change, Agriculture, and Food Security. A total of 630 farm households from three districts of Bangladesh were interviewed (Table 1).

**Table 1:** Distribution of the sampled households

Khulna division						Barisal division	
Satkhira district				Bagerhat district		Jhalokathi district	
Village	*n*	Village	*n*	Village	*n*	Village	*n*
Burigoalini	45	Hatsala	28	Gabgasia	66	Boro Galua + Durgapur^a^	40
Chandipur	66	Sreefal Khati	45	Joka	40	Gopalapur	32
Dumuria	64	Horinagor	45	Teligati	50	Jagannathpur	64
						Tarabunia	45
Total	175		118		156		181

^a^Due to the small sample size, we include two villages together

A multistage sampling method was applied to select households. First, the three districts were purposefully chosen due to their vulnerability to climate associated risks. Second, six villages from Satkhira district, three villages from Bagerhat district, and five villages from Jhalokathi district were selected as study villages (Table 1). Individual households were then sampled randomly.

Household survey was carried using a structured questionnaire. Information on farm household characteristics (i.e., family size, age distribution, educational level, gender of household head, etc.), economic and social capital related variables (i.e., land holdings, livestock owned, household assets, credit and market access, membership in farmers’ groups, food security status, and contact with non-governmental organizations (NGOs) or extension, and access to training) and perceptions of the major climate risks faced and the adaptation strategies applied by households were collected.

In addition, four follow-up focus group discussions (FGDs) were carried out in two villages of Satkhira district to understand local gender norms, surveyed farmers’ perceptions of climate risks and adaptation strategies used in their communities. The FGD was carried out separately for men and women farmers to capture gender-differentiated perceptions of the impacts of climate risks and the use of adaptation strategies.

## Conceptual Framework, Analytical Methods, and Estimation Issues

Climate risks, including extreme weather events, can adversely affect agricultural production and hence, the livelihood of farm households (Eitzinger et al. 2018; IPCC 2014). To reduce the adverse impacts of climate risks on their livelihoods, farmers will consequently apply a number of adaptation strategies (Amare and Simane 2017), which may or may not be similarly linked to other livelihood pursuits. Adaptation to the impact of climate change, in principle, can be planned for short-, medium-, and long-term. Short-term adaptation measures involve urgent response measures to prevent or mitigate the impact of climate change that are already occurring or likely to arise. Examples include the use of savings, borrowing from others, or depending upon government and nongovernmental aids. Medium-term adaptation measures are those designed to mitigate possible impacts of climate change that may occur in the medium or long term in order to reduce vulnerabilities and to strengthen resilience. Example of such medium-term adaptation strategies includes a change in cropping practices, seed replacement, varietal change, etc. Long-term adaptation strategies include the practices designed to cope with climatic impacts in the long-run such as construction and functional improvements of embankments to cope with sea-level rise and storm surges, construction of irrigation channel to cope with long-term drought induced by climate change and so on. Most of the adaptation strategies reported by farmers are short- to medium-term strategies. However, we did not necessarily distinguish farmers’ adaptation strategies into short-, medium-, and long-term and presented them as an individual strategy.

**Fig. 2:**
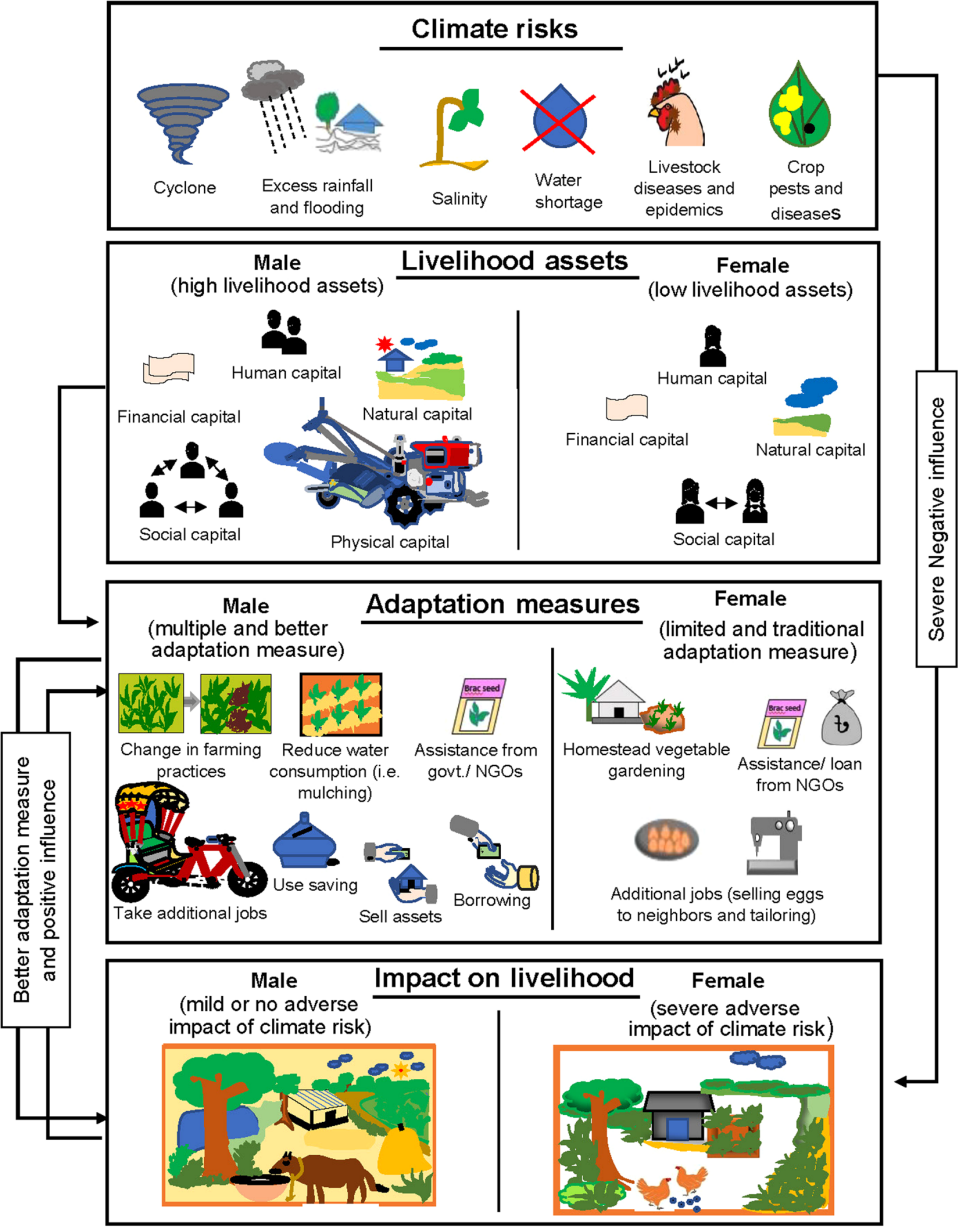
Farm household’s adaptation strategy to the impact of climate change: conceptual framework applied in this study

The decision to adopt particular adaptation strategies can be influenced by farmers’ perceptions of the overall impact of such risks on their livelihoods on the one hand, and the capacity of the household to adapt and adopt such strategies on the other (Fig. 2). Figure shows that the climate risk is covariate shock s and affects all the households; however, the household ability/capacity to manage and cope with the climate risk are influenced by the livelihood assets possessed by the households. The household with more livelihood assets are able to adopt adaptation strategies such that the adverse impact is minimized. The figure also shows the gender-differentiated impact of the climate change risk owning to the command over the livelihood assets. Females have low livelihood assets and hence, limited capacity to cope with the climate risk and are more vulnerable to climate shocks.

Household’s capacity to make use of adaptation strategies largely depends on the level of livelihood assets possessed by the household (Berman et al. 2015; Jakobsen 2013). Livelihood assets consist of human, natural, financial, physical, and social capital (Ellis 2000), which influences the household’s ability and capacity to adopt the adaptation strategies to cope and manage climate risk. For a household, human capital refers to the amount and the quality of family labor, including education and training. Though natural capital implies access to natural resources, including soils, biodiversity, and quality water, we include land assets as this was the simplest variable for farmers to understand and respond to in surveys. In financial capital, we include access to credit, and a household asset index. We used asset index rather than household income in our analysis as the latter is generally either under or over reported in surveys (Booysen et al. 2008). Social capital refers to social resources that enable farm households to pursue their livelihood objectives. We also considered gender to be a conditioning factor influencing social capital as it can define individual’s level of participation and role in social activities in the public sphere, for example including being an active member of cooperatives, village institutions, or having access to financial support (Aryal et al. 2014, 2018; Doss 2018; World Bank 2011). For example, membership in village organizations can be one of the key pillars of social capital that enhances social networks and connectedness with other community members to build relationships of trust, reciprocity, and exchange, each of which may play a role in adaptation to climatic risks (Mersha and Van Laerhoven 2016). Besides this, in the context of Bangladesh, specific gender inequalities and discriminations add to women’s vulnerability to climate extremes (Corcoran-Nantes and Roy 2018; Jordan 2019; Reggers 2019). Moreover, gender differences in Bangladesh are highly observed in the access to and the ownership of all the livelihood assets in general, and particularly, to the ownership of land. On average, women’s command over livelihood assets is lower, and thus, the impact of climate risks on their livelihood is supposed to be worse compared with men (Ministry of Environment and Forest 2013). This eventually can influence their choice of climate adaptation strategies (Fig. 2).

Based on these issues, we address the following hypotheses. (i) Households that experience a higher level of “damage,” in terms of the effects of climate risks on their livelihoods, are more likely to adopt adaptation strategies compared with other households. (ii) Households with more livelihood assets, when experience with a higher level of damage due to climate risks, are more likely to apply adaptation strategies compared with other households. (iii) Households that experience more damage from extreme climate events are more likely to undertake multiple adaptation strategies than those experiencing less damage. (iv) Finally, male-headed households are more likely to apply multiple adaptation strategies as compared with female-headed households.

As noted in the fourth hypothesis, to adapt to the impact of climate change, a farm household may apply multiple strategies. In the cases when the severity of climate risks becomes very high, their own efforts may not be sufficient to effectively manage risks. Under such circumstances, farmers may seek external assistance from governmental or nongovernmental and civil society organizations. Once the adverse effect of climate risks on their livelihood crosses a self-determined threshold, changing farm practices, reducing consumption, or using savings and borrowings alone cannot improve their situation (personal communication in FGDs). Under these circumstances, in our conceptual framework, farmers can, therefore, opt to make use of both farming and nonfarming strategies.

### Analytical Methods

#### Binary logit model

In our conceptual framework, we consider farmers’ decision to adopt certain adaptation strategies as a series of discrete choices. Assume that *Y* denotes the decision to adopt at least one climate adaptation strategy by the farmer, and thus, *Y*∈{0,1}, where *Y* = 1 if farm household adopts climate adaptation strategies and 0 otherwise. The probability of a farm household adopting climate risk mitigating strategies [Pr(*Y* = 1)] is consequently derived using Eq. (1):

1 $${\mathrm{Pr}}\left( {{\it{Y}} = 1} \right) = \Phi \left[ {\mathop {\sum}_{{\it{k}} = 1}^{\it{K}} {\beta _{\it{k}}{\it{X}}_{\it{k}}} } \right].$$

Since the response is a binary outcome, the two events derived from disjoint sets are complementary, and the probability associated with the alternative result (adopting climate adaptation strategies) can be represented by Eq. (2):

2 $${\mathrm{Pr}}\left( {{\it{Y}} = 0} \right) = 1 - \Phi \left[ {\mathop {\sum}_{{\it{k}} = 1}^{\it{K}} {\beta _{\it{k}}{\it{X}}_{\it{k}}} } \right].$$

Taking the partial derivative of the above equation considering the explanatory variable *X*_*K*_, the marginal effect is obtained from Eq. (3):

3 $$\frac{{\partial {\mathrm{Pr}}({\it{Y}} = 1)}}{{\partial {\it{X}}_{\it{k}}}} = \Phi \left[ {\mathop {\sum}_{{\it{k}} = 1}^{\it{K}} {\beta _{\it{k}}{\it{X}}_{\it{k}}} } \right] \times \beta _{\it{k}}.$$

The probability of the *i*^th^ farm household adopting climate adaptation strategies, *P*_*i*_ = *P*(*Y*_*i*_ = 1), therefore depends on a set of explanatory variables, *X*_*i*1_, …….., *X*_*ik*_, as described in Eq. (4), with *ε* indicating random error.

4 $${\it{Y}}_i = \alpha + \beta {\it{X}}_{\it{i}} + \varepsilon.$$

#### Multivariate probit

To assess the determinants of the choice of multiple climate adaptation strategies by the farmers, we applied a multivariate probit model. Univariate logit and probit models are not appropriate in this case and may generate biased estimates as they are based on the assumption of the independence of error terms of the different adaptation strategies implemented by farmers. Univariate techniques could also exclude crucial information about interdependent and simultaneous adoption decisions (Greene 2019). A farm household is more likely to apply multiple adaptation strategies simultaneously to reduce the effects of climate risks on their livelihoods. Therefore, it is highly likely that the decision to adopt one strategy can influence the adoption of multiple other strategies. In such a case, applying a multivariate probit model, we acknowledge dependencies between decisions and the potential correlation among unobserved noise in our equations, while also obtaining unbiased and efficient estimates (Greene 2019; Wooldridge 2012). This also reduces the possibility of observing limited adoption of one or more adaptation strategies due to the nonadoption of other complementary strategies. Nevertheless, without effectively correcting for these complementarities, we would not be able to account for these issues. That is why the decision to adopt risk coping strategies is inherently multivariate.

The multivariate probit model helps us to determine possible complementarities (positive correlation) and substitutability (negative correlation) between adaptation strategies employed by the farmers. From the survey data of the study area, we identify that farm households commonly adopt five major types of climate adaptation strategies, i.e. (i) changing farming practices, (ii) use of savings and borrowings, (iii) a reduction in household consumption, (iv) seek off-farm or other farm laborer employment options, (v) take assistance from government, and/or (vi) NGOs.

A farmer is more likely to adopt a particular climate adaptation strategy if the benefit from its adoption is higher than nonadoption. Consider the *i*^th^ farm household (*i=* 1,2,…,*N*) facing a decision on whether to adopt the *j*^th^ climate adaptation strategies (where *j* denotes a choice of: changing farming practices (*F*), using savings and borrowings (*S*), reducing household consumption (*C*), seeking off-farm or other farm laborer employment options (*J*), take assistance from government (*G*), and/or from NGOs (*N*)). Let *U*_0_and *U*_*j*_ represent the benefits to a farmer without and with the adoption of climate adaptation strategies. A farmer will decide to adopt the *j*^th^ climate adaptation strategies if the net benefit $\left( {{\it{B}}_{{\it{ij}}}^ \ast } \right)$ of its adoption is higher than without it, i.e., ${\it{B}}_{{\it{ij}}}^ \ast = {\it{U}}_{\it{j}}^ \ast - {\it{U}}_0\,\,\, > \,0$. In this case, the net benefit of adopting a climate adaptation strategy is a latent variable, which is determined by household capital endowments, and location characteristics (*X*_*ij*_) and the error term (ε_*ij*_) as in Eq. (5):

5 $${\it{B}}_{{\it{ij}}}^ \ast = {\it{X}}^\prime _{{\it{ij}}}{\it{\upbeta }}_{\it{j}} + \varepsilon _{{\it{ij}}}\,\left( {{\it{F,}}\,{\it{A,}}\,{\it{J,}}\,{\it{G,}}\,{\it{N}}} \right).$$

Equation 5 can be presented in terms of an indicator function. In this case, the unobserved preferences in Eq. 5 translate into the observed binary outcome equation for each adaptation strategy choice, as shown in Eq. (6):

6 $${\it{B}}_{{\it{ij}}}^ \ast = \left\{ {\begin{array}{*{20}{c}} {1\,\,\,\,{\mathrm{if}}\,{\mathrm{B}}_{{\it{ij}}}^ \ast \ge 0} \\ {0\,\,\,{\mathrm{otherwise}}} \end{array}} \right..$$

In the multivariate probit model with the possibility of adopting multiple climate adaptation strategies, the error terms jointly follow a multivariate normal distribution with zero conditional mean and variance normalized to unity, i.e., ${\it{u}}_{\it{F}}{\mathrm{,}}\,{\it{u}}_{\it{A}}{\mathrm{,}}\,{\it{u}}_{\it{J}}{\mathrm{,}}\,{\it{u}}_{\it{G}}{\mathrm{,}}\,{\it{u}}_{\it{N}}\,\mathop{\longrightarrow}\limits^{{MVN}}\left( {0,\,\omega } \right)$. The resulting covariance matrix (*ω*) is given by Eq. (7):

7 $$\omega = \left[ {\begin{array}{*{20}{c}} 1 & {\mathop {\rho }\nolimits_{{\it{FA}}} } & {\mathop {\rho }\nolimits_{{\it{FJ}}} } & {\mathop {\rho }\nolimits_{{\it{FG}}} } & {\mathop {\rho }\nolimits_{{\it{FN}}} } \\ {\mathop {\rho }\nolimits_{{\it{AF}}} } & 1 & {\mathop {\rho }\nolimits_{{\it{AJ}}} } & {\mathop {\rho }\nolimits_{{\it{AG}}} } & {\mathop {\rho }\nolimits_{{\it{AN}}} } \\ {\mathop {\rho }\nolimits_{{\it{JF}}} } & {\mathop {\rho }\nolimits_{{\it{JA}}} } & 1 & {\mathop {\rho }\nolimits_{{\it{JG}}} } & {\mathop {\rho }\nolimits_{{\it{JN}}} } \\ {\mathop {\rho }\nolimits_{{\it{GF}}} } & {\mathop {\rho }\nolimits_{{\it{GA}}} } & {\mathop {\rho }\nolimits_{{\it{GJ}}} } & 1 & {\mathop {\rho }\nolimits_{{\it{GN}}} } \\ {\mathop {\rho }\nolimits_{{\it{NF}}} } & {\mathop {\rho }\nolimits_{{\it{NA}}} } & {\mathop {\rho }\nolimits_{{\it{NJ}}} } & {\mathop {\rho }\nolimits_{{\it{NG}}} } & 1 \end{array}} \right],$$

where *ρ* denotes the pairwise correlation coefficient of the error terms corresponding to any two climate adaptation strategies. If these correlations in the off-diagonal elements in the covariance matrix become nonzero, it justifies the application of a multivariate probit instead of a univariate probit for each individual climate adaptation strategy.

#### Follow-up focus group discussions

Four FGDs were carried out with local farmers in Satkhira district to elucidate the preliminary survey results. Each FGD consisted of 10–15 farmers previously surveyed, including key informants for each study area. A detailed checklist was used for discussions with participants, which was supported by local researchers experienced in facilitating FDGs. The information collected in FGDs was used to validate and complement the results obtained through the multivariate probit model. This provided a more nuanced understanding of climate risks in the study area and also the adaptation strategies commonly implemented by farmers. FGDs with male and female farmers were conducted separately by male and female facilitators, respectively, to provide an indication of why there might be differences in the choice of adaptation measures applied by men and women farmers.

## Results and Discussion

### Descriptive Results

Table 2 presents major climate risks that farmers in southwest coastal Bangladesh faced during the last five years prior to the survey (from January 2008 onwards), as well as adaptation strategies applied by them. Cyclones, excessive rains and flooding, and soil and water salinity were listed as three of the key climate risks. Increased livestock diseases and crop pests and diseases were also associated with the climate and were indicated as increasing risks to rural livelihoods. We considered both direct and indirect climatic risks in subsequent analyses.

**Table 2:** Major climate risks faced by households (*n* = 630) and the adaptation strategies

	Percent of househsolds^a^
Climate-related risks	
Cyclones	90
Soil and water salinity	45
Extreme rain events, storms, and flooding	26
Crop pests and diseases	22
Livestock diseases	23
Climate adaptation strategies	
Change in farming practices^b^	29
Use savings or borrowing of money^c^	42
Reduce household consumption^d^	26
Seek off-farm or other farm laborer employment options^e^	22
Seek assistance from the government	24
Seek assistance from NGOs	18

^a^Multiple responses were observed

^b^Change in farming practices includes replanting damaged crops, follow better fertilizer management practices, crop protection, livestock replacement, and crop varietal or rotational diversity, shifting to new fields, and substituting crops with livestock

^c^Use of savings or borrowing money includes selling assets (i.e., livestock, land, and jewelry or household goods) to buy food from markest

^d^Reduced household consumption includes eating less (reducing meal quantity/quality) and spending less household money on nonfood items

^e^Includes both in the agricultural (farm laborer) and nonagricultural sectors

Cyclones were strongly indicated as the predominant climate risk faced by the 90% of the farmers in the study area, followed by soil and water salinity (45%) and excessive rain and storms and flooding (26%). Of the indirect climatic risks, livestock diseases and epidemics and crop pests and diseases were reported by 23% and 22% of respondents, respectively. Farmers have used several agricultural, economic, and other measures to adapt to these climate risks (Table 4), which can be structured into six major adaptation strategies for the analysis. Use of savings and borrowings was, for example, the most prominent strategy adopted by 42% of households to cope with climate-related production and farm damage risks, followed by changes in farming practices (29%), reduced household consumption (26%), and seeking assistance from the government (24%) and/or NGOs (18%). Twenty-two percent of surveyed farmers also sought off-farm or other farm laborer employment options to mitigate their household’s exposure to income disrupting climate risks.

Ninety percent of the sampled households are male-headed. Only 10% are headed by females (Table 3). The average age of the head of sampled households was 47 years, with 21 years of experience. The majority of sampled household heads were literate, which signifies that majority of the households may have knowledge of climate change and its risks. Nevertheless, only 7% of the total respondents had received any education and/or training on climate change related issues.

**Table 3:** Descriptive statistics of explanatory variables used in the analysis

Variables	Mean	Standard deviation	Variable description
Male-headed HH (D)	0.90	0.30	1 if male headed HH and 0 if female headed HH
Age of HH head	47	13	Age of HH head (years)
Farming experience	20.89	13.29	Years of experience in farming
Literate HH head (D)	0.71	0.45	1 if HH went to school and 0 otherwise
HH labor	3.27	1.27	HH labor availability (adult equivalents)
Training (D)	0.07	0.18	1 if HH members have received formal or informal education on climate change
Farm size	0.44	0.48	Total farm land operated (ha)
Livestock herd size	0.92	1.19	Livestock owned (tropical livestock units)
Credit access (D)	0.69	0.46	1 if HH has credit access and 0 otherwise
Membership (D)	0.39	0.49	1 if HH is member in village institutions including cooperatives/farmers groups and 0 otherwise
Asset index^a^	0.43	0.75	Household asset index
Distance to market	3.54	4.02	Distance to nearest main market from the house (km)
Livelihood effects (categorical)			
No effects on livelihood	0.02		0 if there are no effects of climate on livelihoods
Low effects on livelihood	0.08		1 if livelihood effect is up to 40%
Medium effects on livelihood	0.41		2 if livelihood effect is more than 40% and up to 60%
High effects on livelihood	0.49		3 if livelihood effect is more than 60%

*HH* households, *D* dummy variable

^a^To capture the effect of wealth on the choice of risk coping strategies, we constructed household asset index using principal component analysis (for details, see https://www.stata.com/manuals13/mvpca.pdf). We included most of the household assets such as tractor, car, television, water pump, motorbike, etc. for constructing household asset index

We also analyzed the perception of the effect of the climate risk on farmers’ livelihoods and found that 49% of the sampled households reported that climate-related risks had a high degree of influence on their livelihoods and 41% reported that the effect was medium, and only 8% reported that the effect was low. Only 2% reported no effect.

Majority of the farmers in the study area were smallholders with the average farm size of 0.44 ha (Table 3). About 69% of the sampled households were able to access financial credit. Household memberships in cooperatives and farmers’ groups were about 39%, reflective of Bangladesh’s active civil society. The asset index of the sampled household was 0.43, and the average distance to the major market at which farm produce could be traded from the household was 3.54 km.

### Econometric Results

#### Factors influencing the decision to adopt climate adaptation strategies

Table 4 presents the analysis of factors affecting the decision to adopt at least one adaptation strategy by the farm household. The dependent variable is binary in nature (1 if a household adopted atleast one; 0 otherwise); hence, the logit model was the most suitable estimation method. For the ease of interpretation, we reported only the marginal effects of the explanatory variables on the decision to adopt a climate adaptation strategy. The Wald Chi-square test of the model specification is significant at 1% level, indicating that the valid model specification.

**Table 4:** Logit model marginal effects of the factors influencing farmer household (HH) decisions to adopt climate adaptation strategies

Explanatory variables	Marginal effects on the decision to adopt climate adaptation strategies
Male-headed household (D)	0.130*** (0.018)
Years of farming experience of the HH head	0.003 (0.007)
Literate HH head (D)	−0.008 (0.035)
Credit access (D)	−0.016 (0.039)
Membership in farmers’ groups or cooperatives (D)	0.049** (0.023)
Livestock (in tropical livestock units)	0.012** (0.005)
Land owned (in ha)	0.031*** (0.012)
Asset index	0.197*** (0.074)
Prior education/training on climate change (D)	0.155*** (0.057)
Low effect on livelihood (D): base category “no effect”	−0.169 (0.161)
Medium effect on livelihood (D): base category “no effect”	0.164*** (0.030)
High effect on livelihood (D): base category “no effect”	0.192*** (0.042)
Satkhira district (D): base category “Jhalokathi district”	0.177*** (0.043)
Bagerhat district (D): base category “Jhalokathi district”	−0.036 (0.044)
Log pseudolikelihood	−758.626
Pseudo *R*-squared	0.260
Wald chi-square (14)	68.09
Probability > chi-square	0.000
Number of observations	630

Standard errors are reported in the parentheses

*D* dummy variable

*, **, and *** are significant at the *p* < 0.10, *p* < 0.05, and *p* < 0.01 level, respectively

Our results showed that households’ capital assets play a crucial role in making the decision to adopt a climate adaptation strategy. The gender of the head of the family is found to significantly influence the household decision on whether or not to adopt any climate adaptation strategies. Male-headed households are 13% more likely to adopt at least one adaptation strategy compared with those headed by women (Table 4). In contrast to our a priori assumption that households with more years of farming experience are more likely to adopt adaptation strategy, our data did not indicate a relationship between years of farming and the likelihood of adoption of climate adaptation strategies. The coefficient of the membership in a cooperative and/or farmers’ organization is positive and significant, implying that social capital, for example, participation in civil society groups and community organizations, is an important element in enhancing climate change adaptation.

Similarly, our results showed that capital assets, including livestock, land, and other assets indicative of wealth, were positively associated with the household’s likelihood of adopting climate adaptation strategies, probably due to increased investment capability.

Empirical results showed that households that perceived medium- and high-level effects of climate risks on their livelihood were more likely to adopt climate adaptation strategies when compared with households with no adverse effect of climate risks on their livelihoods.

#### Factors influencing farmers’ choice of the type of adaptation strategies

We identified six commonly adopted measures used by farm households to adapt to climate risks. These included change in farming practices, use past savings or borrowing money, reducing household consumption, seek off-farm or other farm laborer employment options, and taking assistance from the government and/or NGOs. As the six adaptation strategies can be mutually inclusive, i.e., the use of one measure may not preclude the use of another; we utilized a multivariate probit model to determine the factors influencing farmers’ choice of adaptation strategies. Our estimation of the correlation of error terms of selected climate adaptation measures supports the suitability of the multivariate probit model (Table 5). Many pair-wise correlation coefficients of the residuals of climate adaptation strategies are found to be statistically significant, implying that the error terms in selection decisions of multiple climate adaptation strategies are correlated. This justifies the use of a multivariate probit model instead of using an independent probit or logit model in the analysis of farm household’s adoption of climate risk mitigation strategies. We find that households who changed their farming practices were less likely to adopt other adaptation strategies. Households make use of their savings and/or borrow money to adapt with climate risk are also more likely to seek additional employment. Households that adapt to climate risks by reducing consumption are conversely less likely to seek additional income generation options, and are more likely to take assistance from NGOs. Farm households that have members taking additional jobs are also more likely to take assistance from the government. It is also interesting to note that households who take assistance from NGOs are also less likely to take assistance from the government. These results indicate a need for collaboration between governmental and NGOs in order to better support poor household to mitigate the adverse impact of climate risks.

**Table 5:** Correlation of error terms of selected climate adaptation measures

Correlation pairs	Coefficient	Standard error
“Change in farming practices” and “use past savings or borrowing money”	−0.248**	(0.118)
“Change in farming practices” and “reduce household consumption”	−0.093	(0.070)
“Change in farming practices” and “seek off-farm or other employment”	−0.418***	(0.080)
“Change in farming practices” and “take assistance from government”	−0.336***	(0.066)
“Change in farming practices” and “take assistance from NGOS”	−0.018	(0.060)
“Use past savings or borrowing money” and “reduce consumption”	−0.038	(0.086)
“Use past savings or borrowing money” and “take additional jobs”	0.118**	(0.057)
“Use past savings or borrowing money” and “take assistance from government”	0.056	(0.081)
“Use past savings or borrowing money” and “take assistance from NGO”	−0.093	(0.081)
“Reduce household consumption” and “take additional jobs”	−0.148**	(0.070)
“Reduce household consumption” and “take assistance from government”	0.104	(0.065)
“Reduce household consumption” and “take assistance from NGOs”	0.394***	(0.062)
“Seek off-farm or other employment” and “take assistance from government”	0.189***	(0.062)
“Seek off-farm or other employment” “take assistance from NGOs”	−0.039	(0.064)
“Seek off-farm or other employment” and “take assistance from government”	−0.082**	(0.035)

Likelihood ratio test of rho21 = rho31 = rho41 = rho51 = rho61 = rho32 = rho42 = rho52 = rho62 = rho43 = rho53 = rho63 = rho54 = rho64 = rho65 = 0; chi2 (15) = 169.574; probability > chi-square = 0.0000

*, **, and *** are significant at *p* < 0.10, *p* < 0.05, and *p* < 0.01 level, respectively

Our analyses indicate that the gender of the household head emerges as an important driver of the choice of climate adaptation strategies (Table 6). Male-headed households are more likely to change the farming practices, take additional jobs, reduce consumption, and take assistance from the government while they are less likely to take assistance from NGOs. In recent years, many NGOs actively target female-headed households as development project beneficiaries in comparison with governmental organizations. Farmers that participated in the FGDs also reported that female farmers tended to get more assistance from NGOs for vegetable farming and other income-generating activities that were also referenced as climate adaptation strategies.

**Table 6:** Analysis of factors associated with the choice of climate adaptation strategies by the farmers (results of multivariate probit model)

	Climate adaptation strategies					
Explanatory variables	Change in farming practices	Use past saving/borrowing	Reduce consumption	Take additional job	Take assistance from government	Take assistance from NGO
Male-headed HHs	0.440** (0.213)	0.418 (0.319)	0.243** (0.117)	0.196** (0.091)	0.656*** (0.241)	−0.272*** (0.103)
Farming experience	0.093*** (0.025)	0.137 (0.158)	0.156*** (0.044)	−0.204*** (0.058)	−0.042 (0.040)	−0.049 (0.040)
Literate HH head	0.115 (0.102)	−0.089 (0.169)	−0.148 (0.108)	0.166*** (0.053)	0.159** (0.069)	0.022 (0.100)
Credit access	0.165 (0.114)	0.211*** (0.078)	0.057 (0.123)	−0.174 (0.117)	0.388*** (0.115)	−0.102 (0.111)
Membership	0.121* (0.065)	0.030 (0.178)	−0.134 (0.115)	0.159 (0.107)	−0.083 (0.096)	0.114*** (0.042)
Livestock	0.067* (0.038)	0.137** (0.058)	0.013 (0.044)	−0.204*** (0.058)	−0.042 (0.040)	−0.049 (0.040)
Land owned	0.110*** (0.039)	0.376*** (0.152)	0.165 (0.103)	−0.359** (0.152)	0.113 (0.087)	0.189** (0.089)
Asset index	0.017 (0.103)	0.189** (0.083)	−0.116*** (0.046)	−0.178* (0.103)	0.215*** (0.083)	−0.223** (0.106)
Training	0.397*** (0.104)	−0.756 (0.564)	−0.858* (0.452)	0.635*** (0.228)	0.338* (0.203)	−0.306 (0.261)
Low effect on livelihood	−0.242 (0.298)	0.107 (0.561)	−0.165 (0.403)	−0.041 (0.545)	−0.098 (0.368)	−0.495 (0.338)
Medium effect on livelihood	−0.595** (0.267)	0.064*** (0.027)	−0.102*** (0.036)	0.620* (0.346)	0.030 (0.338)	0.247*** (0.089)
High effect on livelihood	−0.739*** (0.267)	0.273*** (0.101)	−0.221*** (0.042)	0.684 (0.485)	0.112*** (0.037)	0.275** (0.128)
Satkhira district	0.512*** (0.146)	0.213 (0.239)	0.985*** (0.216)	0.256* (0.149)	−0.078 (0.123)	0.567*** (0.142)
Bagerhat district	0.257 (0.168)	0.066 (0.286)	0.092 (0.264)	−0.477** (0.207)	0.163 (0.139)	−0.265 (0.193)
Constant	−1.690*** (0.376)	−5.851** (2.620)	−2.405*** (0.463)	−1.598*** (0.537)	−2.144*** (0.430)	−1.785*** (0.389)
Log likelihood	−2762.72					
Wald chi-square (84)	347.60					
Probability > chi-square	0.000					
No. of observations	630					

Standard errors are reported in parentheses

*, **, and *** refer to significant at the *p* < 0.10, *p* < 0.05, and *p* < 0.01 level, respectively

The number of years in farming also appears to influence the choice of climate adaptation strategies: more experienced farmers in our survey were more likely to flexibly opt to change farming practices and reduce household consumption. They were also less likely to seek additional income generating opportunities through agricultural or nonfarm employment. As experienced farmers have greater knowledge about farming, it is easier for them to change the farming practices, and it is also more likely that they have limited skills set to look for alternative employment.

Households with literate head tend to take additional jobs. This result is not surprising as literate individuals tend to have knowledge and skills that enable them to envision nonfarm opportunities as viable, and will be more likely to seek alternative employment opportunities. Literate household heads also appear to seek assistance from the government more than those who are not.

Households with access to credit positively influence their use of past savings/borrowing money and also taking assistance from the government as climate adaptation strategies. Membership in cooperatives and farmers’ associations was also positively associated with households’ ability to change farming practices, and to access assistance from NGOs that often target working with farmers’ organizations in implementing development projects.

The households with more livestock and land assets are also more likely to change farming practice, use savings or to borrow money, and are less likely to seek additional employment to mitigate the adverse impact of climate risks on their livelihoods. More livestock and land assets indicate that these households are relatively wealthy and are less likely to look outside the farm for additional income. Households with more land are also positively associated with assistance from the government. The household assets index is positively associated with the use of savings or borrowing money, although households that are likely to seek assistance from the government are less likely to resort to reducing household consumption, seek additional employment and income sources, or to take assistance from NGOs. Farmers who participated in FGD reported that they did not receive sufficient aid from the government even though a large amount of funding was allocated for climate risk relief. Beneficiaries of such allocations were mostly relatively rich and influential people in the village. The participants in FGD asked for developing appropriate policy and monitoring mechanisms so that the poor and marginal farmers get adequate support to cope with climate risks.

Farmers who had engaged in educational training and who had become aware of climate change were positively associated with a willingness to change their farming practice and seek additional employment options. Training on climate change was also negatively associated with reduced household consumption, indicating that households with more understanding of the mechanisms that affect climate change and their consequent results were less likely to opt for drastic food consumption reduction adaptation measures.

The level of perceived impact of climate risks on livelihoods also exerts a significant influence on the uptake of adaptation strategies. Households with perceptions that impacts of climate risks on livelihoods are minimal do not adopt climate adaptation strategies. Households with perceptions of medium and high levels of negative effects mostly tend to use past saving/borrowing, additional jobs, assistance from government, and NGOs to adapt with climate risks. We also noted spatial variation on the climate adaptation strategies adopted by the household.

## Discussion and Policy Implications

Our empirical findings corroborate with the findings of previous researches on climate change adaptation and gender. In support of the literature on stark gender differences in farm management in coastal Bangladesh (Akter et al. 2016), our results indicate that the gender of the head of the family significantly influences the household decision on whether or not to adopt any climate adaptation strategies, and also while choosing individual or combination of adaptation strategies. As female-headed households often tend to suffer from labor shortages in Bangladesh (Theis et al. 2019), they are less likely to opt for a change in farming practices as an adaptation strategy (Aryal et al. 2014). Social restrictions on mobility and the burden of household responsibilities, in addition to cultural and social hegemony, prevent many women from seeking an additional job and diversifying their livelihoods (Akter et al. 2016; Aryal et al. 2014, 2019a; Theis et al. 2019). In these cases, differences in the adoption of climate adaptation strategies can also result from inequities in endowments among male and female-headed households (Nabikolo et al. 2012). Large endowment difference between male and female-headed households is possible in Bangladesh because women are mostly involved in unpaid family labor works (Heintz et al. 2018). This has important policy implications related to gender and climate change adaptation in Bangladesh.

Unlike the findings of Abid et al. (2016) and Rahut and Ali (2017) that household with more years of farming experience are more likely to change farming practices to cope with climate risks, we did not find this in our case in Bangladesh.

We found that membership in a cooperative and/or farmers organization is positive and significant, corroborating the findings of others that civil society groups and community organizations are important for climate change adaptation and shared risk-coping in South Asia (Abid et al. 2015; Shikuku et al. 2017). Membership in cooperatives, groups, or similar community structures permit households to integrate into village and regional social networks, allowing families and individuals to learn about and share with each other information regarding new technologies and adaptation practices, which can, in turn, affect their adoption potential (Bandiera and Rasul 2006). However, another study in Vietnam (Trinh et al. 2018) found that membership in organizations did not necessarily influence farm household’s use of climate adaptation strategies. This signifies the context-specific and culturally specific nature of rural and community organizations in climate change adaptation, indicating that the usefulness of group organization to encourage resilience to climate change in agricultural systems should be critically evaluated from location to location. Therefore, development projects in these areas need to focus on increasing poor farmers’ access to farm cooperatives and farmers association as a way to enhance their ability to implement climate change adaptation strategy.

Like other studies (Abid et al. 2020; Bryan et al. 2009; Rahut and Ali 2018), our results showed that capital assets including livestock, land, and other assets indicative of wealth were positively associated with the household’s likelihood of adopting climate adaptation strategies, probably due to increased investment capability. These results indicate the importance of development interventions and policies to enable poor also to adapt to climate change.

Despite the fact that most of the farmers had medium to high-level effects of climate risks on their livelihoods, only resource-rich farmers were more likely to adopt climate adaptation strategies. This underscores the need for policy to support adaptation to climate change, particularly to resource-poor farmers, and for female-headed households. We found differences between the districts under study, indicating that there is a variation in the use of climate adaptation strategies by location. Given the increased risks faced by farmers to climate change in different parts of the country, for example in the coastal region where cyclones and extreme weather events are a concern (Aravindakshan et al. 2020), or in north western Bangladesh where drought is more frequent (Qureshi et al. 2015), geographic differentiation of risk is not overly surprising.

Bangladesh’s National Adaptation Programme of Action (NAPA) was developed by the Ministry of Environment and Forests in 2005 and revised in 2009 to acknowledge these issues. Alongside the Bangladesh Climate Change Strategy and Action Plan (Ministry of Environment and Forest 2009), policy actions highlight measures aimed at addressing different physiographic regions within the country, including and with emphasis on the coastal zone. The impacts of climate change on livelihoods and resource-poor farmers are also addressed, but emphasis on measures to reduce negative impacts on women, in particular, are discussed only in relatively basic ways, with limited concrete suggestions for ways in which policies can be aligned to support disadvantaged groups. Our study conversely adds to the growing literature on the importance of considering the gender-differentiated impacts of climate change and consequent livelihood adaptation strategies; as such, it also underscores the need for well-planned and strategic policy to address women’s equity and empowerment. Although the NAPA touches on these issues, our results provide further insights that could, for example, be considered in the formulation of Bangladesh’s eight 5-year plan for 2021–2026, which provides detailed policy and international donor investment guidance to foster national development.

The positive role of education and literacy on the use of climate adaptation strategies across the globe has been confirmed by other research (Abid et al. 2015, 2020; Deressa and Hassan 2009; Deressa et al. 2009; Mohal and MMA 2007; Mulwa et al. 2017; Thomas et al. 2007), suggesting that development initiatives aimed at long-term climate change risk mitigation and that wish to encourage resilience and adaptation strategies could benefit by fundamental efforts to increase literacy and numeracy.

Previous research has shown a positive association between access to credit and climate risk adaptation strategies, although these variables are not always significantly related (Abid et al. 2015; Bryan et al. 2009; Mulwa et al. 2017). Others also confirmed the positive correlation between the adoption of climate adaptation strategies and household assets in Asia and Africa (Bryan et al. 2009; Rahut and Ali 2017). The positive role of training on the adoption of climate adaptation strategies was also found by other research (Mulwa et al. 2017; Trinh et al. 2018). This indicates a need for large-scale education and training programs to increase farmer knowledge on climate change adaptation, which eventually encourages equitable and just development.

In addition, future cross-locational studies can also consider the ways in which organizations and their capacity for adaptation develop and evolve over time, including power differentials within groups (for instance, between men and women farmers), including but not limited to farmers’ groups or associations. Similarly, the role of NGOs in enhancing the capacity of men and women farmers in climate change adaptation, and how to improve the coordination between governmental and NGOs to improve farm households’ capital assets so that climate adaptation is achieved.

## Conclusions

This study explored the major climatic risks in south-western coastal Bangladesh, farmers’ adaptation strategies, and determinants of the choice of those strategies. We found that the livelihood of smallholder farmers in the study area are affected by climatic risks such as cyclones, increasing soil and water salinity, storm surges and heavy rainfall that can result in flooding and waterlogging. Besides, farmers in the study area reported crop/livestock pests and diseases as indirect impacts of climate change.

Major adaptation strategies to reduce the impact of climate change adopted by the farmers in the study area include changes in farming practices, use of savings and borrowing money from others, and reduced household food and goods consumption. In addition, farmers indicated that they may seek to take assistance from the government and/or NGOs, as well as seeking alternative forms of agricultural and nonagricultural employment and income generation.

We found that the choice of adaptation strategy was affected by the gender of the household head, availability of household assets at their disposal, and level of awareness on climate change and its impact. This calls for future policy aimed at increasing the capacity of farmers in South Asia’s climate risk vulnerable coastal areas and should focus on enhancing the knowledge through educational programs, increase access to multiple livelihood opportunities, and information on adaptation strategies, while also working to improve poor and women’s access to resources.

Despite robust results, one of the limitations of this study is that it is based on the cross-sectional data. Therefore, future study with panel data over a long period of time can help in understanding the dynamics of climate change and its impacts on farm households livelihood and also looking deeper into how adaptation strategies evolve over a period of time.
